# Regulatory Effects of Fisetin on Microglial Activation

**DOI:** 10.3390/molecules19078820

**Published:** 2014-06-26

**Authors:** Jing-Yuan Chuang, Pei-Chun Chang, Yi-Chun Shen, Chingju Lin, Cheng-Fang Tsai, Jia-Hong Chen, Wei-Lan Yeh, Ling-Hsuan Wu, Hsiao-Yun Lin, Yu-Shu Liu, Dah-Yuu Lu

**Affiliations:** 1 Department of Medical Laboratory Science and Biotechnology, China Medical University, Taichung 40402, Taiwan; E-Mails: jychuang@mail.cmu.edu.tw (J.-Y.C.); syciac@gmail.com (Y.-C.S.); 2 Department of Bioinformatics, Asia University, Taichung 41354, Taiwan; E-Mail: pcchang@asia.edu.tw; 3 Department of Physiology, School of Medicine, China Medical University, Taichung 40402, Taiwan; E-Mail: clin33@mail.cmu.edu.tw; 4 Department of Biotechnology, Asia University, Taichung 41354, Taiwan; E-Mail: tsaicf@asia.edu.tw; 5 Department of General Surgery, Taichung Tzu Chi Hospital, Buddhist Tzu Chi Medical Foundation, Taichung 42743, Taiwan; E-Mail: guns5150@ms27.hinet.net; 6 Department of Cell and Tissue Engineering, Changhua Christian Hospital, Changhua 500, Taiwan; E-Mail: ibizayeh0816@hotmail.com; 7 Graduate Institute of Basic Medical Science, China Medical University, Taichung 40402, Taiwan; E-Mails: lulu80620@yahoo.com.tw (L.-H.W.); yushuliu220@gmail.com (Y.-S.L.); 8 Department of Life Sciences, National Chung Hsing University, Taichung 402, Taiwan; E-Mail: lingirl831@hotmail.com; 9 Graduate Institute of Neural and Cognitive Sciences, China Medical University, Taichung 40402, Taiwan; 10 Department of Photonics and Communication Engineering at Asia University, Taichung 41354, Taiwan

**Keywords:** microglia, neuroinflammation, neurodegeneration, fisetin, cytokine

## Abstract

Increasing evidence suggests that inflammatory processes in the central nervous system that are mediated by microglial activation play a key role in neurodegeneration. Fisetin, a plant flavonol commonly found in fruits and vegetables, is frequently added to nutritional supplements due to its antioxidant properties. In the present study, treatment with fisetin inhibited microglial cell migration and ROS (reactive oxygen species) production. Treatment with fisetin also effectively inhibited LPS plus IFN-γ-induced nitric oxide (NO) production, and inducible nitric oxide synthase (iNOS) expression in microglial cells. Furthermore, fisetin also reduced expressions of iNOS and NO by stimulation of peptidoglycan, the major component of the Gram-positive bacterium cell wall. Fisetin also inhibited the enhancement of LPS/IFN-γ- or peptidoglycan-induced inflammatory mediator IL (interlukin)-1 β expression. Besides the antioxidative and anti-inflammatory effects of fisetin, our study also elucidates the manner in fisetin-induced an endogenous anti-oxidative enzyme HO (heme oxygenase)-1 expression. Moreover, the regulatory molecular mechanism of fisetin-induced HO-1 expression operates through the PI-3 kinase/AKT and p38 signaling pathways in microglia. Notably, fisetin also significantly attenuated inflammation-related microglial activation and coordination deficit in mice *in vivo*. These findings suggest that fisetin may be a candidate agent for the development of therapies for inflammation-related neurodegenerative diseases.

## 1. Introduction

Microglial activation leads to neuroinflammatory responses that exert both beneficial and detrimental effects, including host defense and tissue repair processes [1,2]. During neuroinflammatory periods, activated microglia are able to clear debris or invading pathogens, and produce neurotrophic factors which modulate the microenvironment [3]. When sensing ATP leaks from an injury site, microglia transform to a more motile state and migrate to the site of damage [4], which causes neuroinflammation and subsequent neurodegeneration. Excessive inflammatory mediators and oxidative stress in the CNS are associated with the pathogenesis of neurodegeneration [5,6]. In pathological neuroinflammatory conditions, microglial activation results in expression of new proteins like inducible nitric oxide synthase (iNOS) which have been shown to cause neuronal damage [7,8,9,10]. It has been reported that iNOS triggers NO production by activated microglia, which further aggravate the pathological processes in neurodegeneration [11]. The expressions of the inflammation-related enzymes iNOS have also been reported in the striatum of Parkinson disease patients [12]. Although inflammatory mediators are necessary for normal neuronal cell functions, the microglial response must be tightly regulated to avoid over-activation and neurotoxic consequences [13]. Interleukin-1 beta (IL-1β) is a major pro-inflammatory cytokine that increases expression of adhesion molecules and synthesis of cytotoxins [14,15]. Microglia activation and neuronal degeneration are co-presence of IL-1β in Alzheimer’s disease [16]. Moreover, IL-1β was observed in cerebrospinal fluid of multiple sclerosis patients, suggesting a possible link between inflammation and neurodegeneration [17].

**Figure 1:**
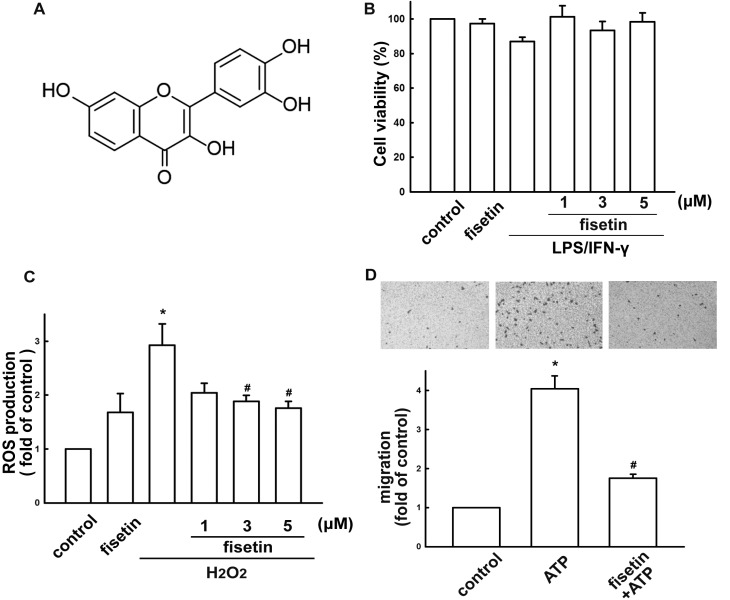
Effect of fisetin on microglial activation. (**A**) Chemical structure of fisetin. (**B**) Cell viability following fisetin treatment in BV-2 microglia. Cells were incubated with concentrations ranging from 1 to 5 μM of fisetin for 60 min followed by treated with LPS (10 ng/mL) plus IFN-γ (10 ng/mL) for 24 h, and cell viability was measured by the MTT assay. The results are expressed as mean ± S.E.M. of three independent experiments. (**C**) Cells were pretreated with various concentrations of fisetin for 60 min followed by stimulation with H_2_O_2_ for 120 min. ROS generation was determined using the fluorescence probes H_2_DCFH-DA and examined by flow cytometry. The results are expressed as mean ± S.E.M. from 3 to 4 independent experiments. * *p* < 0.05 compared with the control group; ^#^ *p* < 0.05 compared with the H_2_O_2_ alone. (**D**) Cells were pre-incubated with various concentrations of fisetin (1–5 μM) for 60 min followed by a 24-h treatment with ATP (300 μM). *In vitro* migratory activities were examined using a cell culture insert system. The results are expressed as mean ± S.E.M. from 3 independent experiments. * *p* < 0.05 compared with the control group; ^#^ *p* < 0.05 compared with the ATP alone. The migrated cells were visualized by phase-contrast imaging (Lower panel).

Many natural products, such as flavonoids, are potential therapeutic agents recognized for their antioxidant activity [18]. Flavonoids are low-molecular-weight natural polyphenolic compounds found in many fruits and vegetables. It has been reported that flavonoids exert many biological functions [19,20]. Fisetin (3,3',4',7-tetrahydroxyflavone; Figure 1A), a high Trolox-equivalent antioxidant, is found in strawberry, persimmon, grape and cucumber [21]. Due to it being a hydrophobic compound, fisetin easily penetrates cell membranes accumulating in cells to exert its antioxidative effects [22], with wide-ranging biological activities including antiaging, anti-inflammatory, neuroprotection and anticancer effects [23,24]. Previous reports have demonstrated the protective effects of fisetin on microglial activation and subsequent neurotoxicity [25]. After oral administration of fisetin could also pass the blood-brain barrier to exert neuroprotective and cognition-enhancing effects in animal model of CNS disorders [26] Importantly, fisetin attenuates postischemic immune cell infiltration and reduces infarct size after transient cerebral middle artery occlusion in mice [27]. Recent study also revealed that orally administered fisetin exerts neuroprotection against reactive gliosis and behavioral deficits in aluminum chloride-induced brain pathology model [28].

Heme oxygenase (HO), a cytoprotective enzyme, degrades heme to bilirubin, carbon monoxide, and iron [29,30]. Induction of HO-1 expression and related signal pathways exert anti-inflammatory effects in macrophages [31,32,33]. Recently, we have reported that neuroinflammatory responses can be repressed by HO-1 induction in microglia [34,35] and astrocytes [36], and that increased HO-1 expression protects neurons against neurotoxin-induced cell death [37,38]. Previous report shown that fisetin protects cells from oxidative-stress-induced death and induces HO-1 in human retinal pigment epithelial cells [39]. Fisetin also been reported to protect against hydrogen peroxide-induced oxidative stress through induction of HO-1 expression in human umbilical vein endothelial cells [40]. A recent study also reported that fisetin up-regulates HO-1 expression and interferes with reactive oxygen species production in macrophage-differentiated osteoclasts [41].

Although the beneficial effects of fisetin in brain have been investigated, the mechanism of regulation of microglia polarity has not yet been determined. In the present study, we addressed whether, in addition to inhibiting cytokine production, HO-1 expression also contributes to fisetin-regulated anti-inflammatory responses in microglial cells.

## 2. Results

### 2.1. Fisetin Suppresses Neuroinflammatory Responses in Microglial Cells

**Figure 2:**
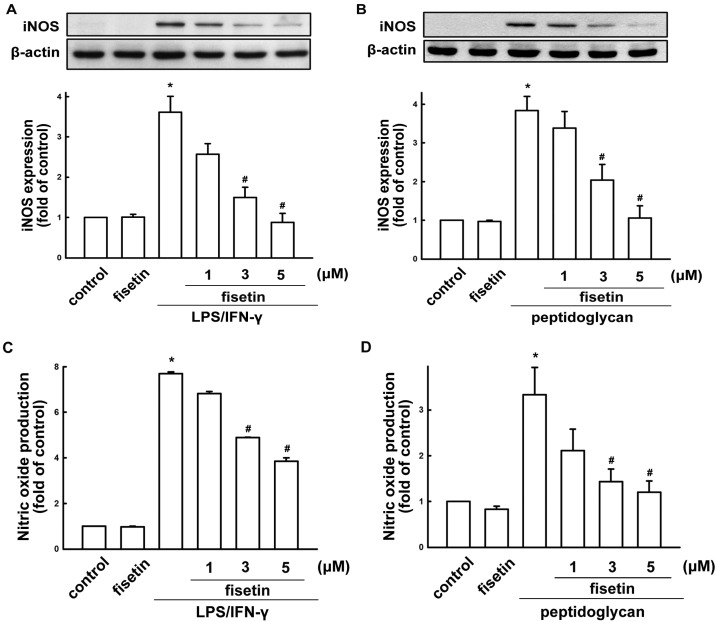
Inhibitory effect of fisetin on LPS/IFN-γ or peptidoglycan-stimulated iNOS/NO expression. (**A**,**C**) BV-2 microglial cells were pretreated with different concentrations of fisetin (1, 3, or 5 μM) for 60 min before application of LPS (10 ng/mL) plus IFN-γ (10 ng/mL) for another 24 h. (**B**,**D**) Cells were pretreated with different concentrations of fisetin (1, 3, or 5 μM) for 60 min before application of peptidoglycan (10 μg/mL) for another 24 h. Western blot analysis for iNOS (**A**,**B**) expression was performed on whole cell lysates. The quantitative results are shown in the bottom panels. The culture media were collected and analyzed NO production by a Griess reaction (**C**,**D**). iNOS or NO expression was significantly different between the LPS/IFN-γ (or peptidoglycan) treated-group and the group treated LPS/IFN-γ (or peptidoglycan) with fisetin. The results are expressed as mean ± S.E.M. from 3 to 4 independent experiments. * *p* < 0.05 compared with the control group; ^#^ *p* < 0.05 compared with the LPS/IFN-γ or peptidoglycan treatment.

We used BV-2 microglia to study the effects of fisetin on neuroinflammatory responses. Concentrations ranging from 1 to 5 µM fisetin were used in the current study. A colorimetric cell viability assay (MTT assay) confirmed that these concentrations did not affect cell viability (Figure 1B). H_2_O_2_ induced an increase in intracellular ROS levels, as shown by H_2_DCF-DA staining which were analyzed by FACS detection assay (Figure 1C). Treatment with fisetin reduced H_2_O_2_-induced ROS productions (Figure 1C). Fisetin inhibited an ATP-induced increase in BV-2 microglial migratory activity (Figure 1D; upper panel). Representative micrographs of migrating cells are shown in Figure 1D (lower panel). To determine the effect of fisetin on iNOS/NO expression, cells were treated with different concentrations of fisetin (1 to 10 µM) and were stimulated with LPS plus IFN-γ. The supernatant of cell culture was then collected to determine NO production. Previously, we have demonstrated that peptidoglycan a major component of the Gram-positive bacterium cell wall, induces neuroinflammatory responses in microglial cells [42,43]. Hence, to further determine the effect of fisetin on nitric oxide production, BV-2 microglia were also stimulated with peptidoglycan. As shown in Figure 2A,B, fisetin effectively inhibited iNOS expression in a concentration-dependent manner following exposure to either LPS (10 ng/mL) plus IFN-γ (10 ng/mL) or peptidoglycan (10 μg/mL). Furthermore, fisetin also reduced LPS/IFN-γ- and peptidoglycan-induced NO production (Figure 2C,D, respectively) in a concentration-dependent manner. 

**Figure 3:**
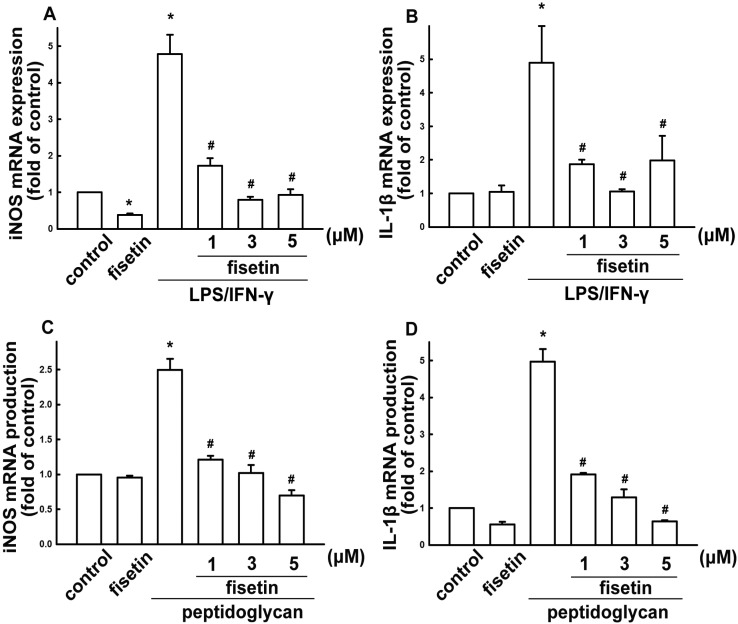
Inhibitory effect of fisetin on LPS/IFN-γ- or peptidoglycan-stimulated iNOS and IL-1β expressions. BV-2 microglial cells were pretreated with different concentrations of fisetin (1, 3, or 5 μM) for 60 min, then challenged with LPS plus IFN-γ (**A**,**B**), or peptidoglycan (**C**,**D**) for another 6 h. The expression of iNOS and IL-1β were determined by real-time PCR. Cytokine expression was significantly different between the LPS/IFN-γ (or peptidoglycan) alone and the LPS/IFN-γ (or peptidoglycan) with fisetin groups. The results are expressed as mean ± S.E.M. from 3 to 4 independent experiments. * *p* < 0.05 compared with the control group; ^#^ *p* < 0.05 compared with the LPS/IFN-γ treatment alone group.

Notably, fisetin treatment alone did not affect iNOS or nitric oxide expression. We further analyzed the expression of inflammatory mediator using real-time PCR. BV-2 microglia were treated with different concentrations of fisetin (1 to 5 µM) and stimulated with LPS plus IFN-γ, or peptidoglycan for 6 h. Fisetin potentiates a concentration-dependent suppression of iNOS when stimulating cells with LPS/IFN-γ or peptidoglycan (Figure 3A,C, respectively). Similarly, treatment with fisetin also inhibited LPS/IFN-γ- or peptidoglycan-induced IL-1β expression in a concentration-dependent manner (Figure 3B,D, respectively). 

**Figure 4:**
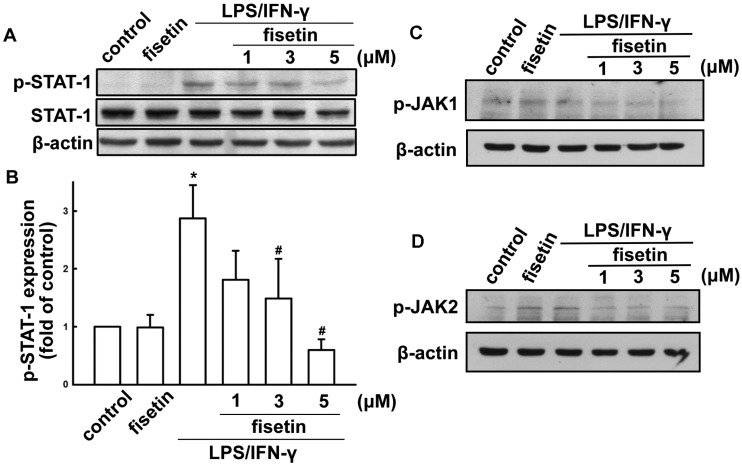
Fisetin suppresses LPS/IFN-γ-induced STAT signaling pathways. BV-2 microglial cells were pretreated with fisetin (1, 3, or 5 μM) for 60 min, then exposed to LPS (10 ng/mL) plus IFN-γ (10 ng/mL) for another 60 min. The phosphorylated levels of STAT-1, JAK1 or JAK2 were determined by western blot analysis, and the signal intensities were normalized to total protein expression. The results are expressed as mean ± S.E.M. from 3 independent experiments. * *p* < 0.05 compared with the vehicle control treatment group. ^#^ *p* < 0.05 compared with the LPS/IFN-γ treatment alone group.

Next, we investigated regulation of STAT-1 protein on activatied effects in microglia. LPS plus IFN-γ treatment resulted in STAT1 (Tyr^7^^0^^1^) phosphorylation (Figure 4A). Treatment with different concentrations of fisetin (1 to 5 µM) attenuated LPS/IFN-γ-induced STAT1 (Tyr^7^^0^^1^) phosphorylation (Figure 4A,B). In addition, fisetin reduced LPS/IFN-γ-induced JAK1 and JAK2 phosphorylation as well (Figure 4C,D, respectively). These results indicate that fisetin exerts anti-inflammatory effects in microglial cells.

### 2.2. Fisetin Induces HO-1 Up-Regulation in Microglia Cells

**Figure 5:**
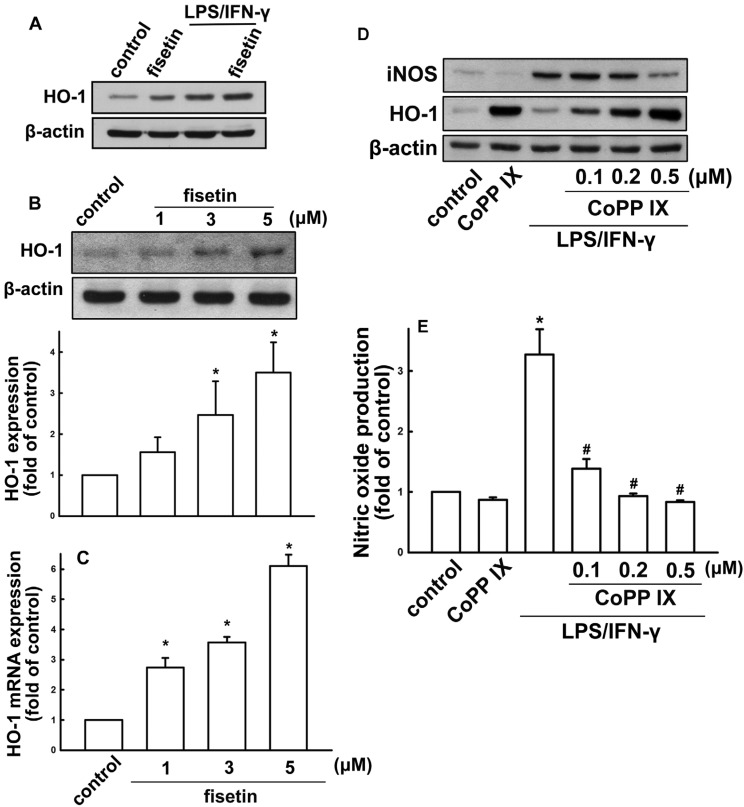
Effect of fisetin on HO-1 expression in microglia cells. (**A**) Cells were treated with fisetin, LPS (10 ng/mL) plus IFN-γ (10 ng/mL), or fisetin with LPS/IFN-γ for 24 h. Cells were treated with different concentrations of fisetin (1, 3, or 5 μM) for 24 h (**B**) or 6 h (**C**). Whole cell lysates were subjected to western blot analysis for HO-1 protein expression (**A**,**B**). HO-1 mRNA expressions were analyzed by real time-PCR (**C**). Cells were treated with Copp IX for 60 min, then challenged with LPS plus IFN-γfor another 24 h. The expression of iNOS and HO-1 were determined by western blot analysis (**D**). NO production was analyzed by Griess reaction (**E**). The results are expressed as mean ± S.E.M. from 3 to 4 independent experiments. * *p* < 0.05 compared with the control group; ^#^ *p* < 0.05 compared with the LPS/IFN-γ treatment alone group.

**Figure 6:**
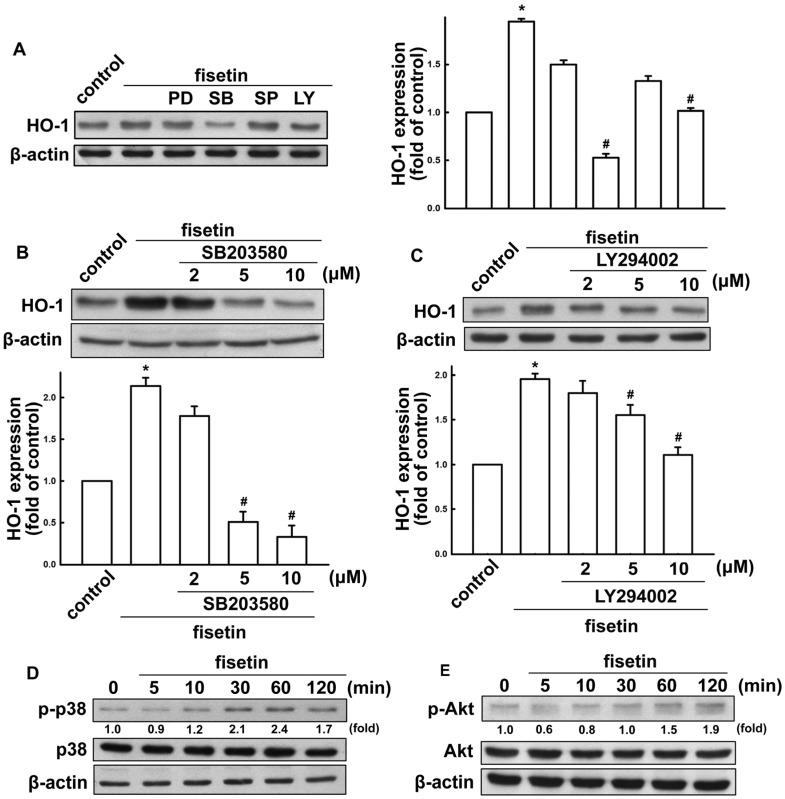
Involvement of p38 and Akt signaling pathways in fisetin-stimulated HO-1 up-regulation in BV-2 microglia. (**A**) Cells were pretreated with various inhibitors (PD98059, SB203580, SP600125 or LY294002) for 60 min, followed by stimulation with fisetin for 24 h. Cells were pretreated with various concentrations of SB203580 (**B**) or LY294002 (**C**) for 60 min, followed by stimulation with fisetin for 24 h. Whole cell lysis proteins was extracted and subjected to western blot analysis for HO-1 expression. Cells were incubated with fisetin for indicated periods (5, 10, 30, 60 or 120 min). Whole cell lysates were subjected to western blot analysis by using antibodies against the phosphorylated p38 (**D**) or Akt (**E**). Similar results were obtained from at least three independent experiments. * *p* < 0.05 compared with the control group; ^#^ *p* < 0.05 compared with the fisetin treatment group.

Since HO-1 induction is known to participate in regulation of inflammatory responses, we investigated whether fisetin might lead to HO-1 induction. When BV-2 microglia were treated with fisetin for 24 h, HO-1 levels increased in a concentration-dependent manner (Figure 5B). In addition, the HO-1 expression was further enhanced under fisetin treatment after LPS/IFN-γ administration (Figure 5A). We further analyzed HO-1 mRNA expression induced by various concentrations of fisetin (Figure 5C). We then used a HO-1 activator CoPP IX to examine whether HO-1 expression participated in the anti-inflammatory pathways. As shown in Figure 5D, treatment with HO-1 activator increased HO-1 expression and abrogated LPS/IFN-γ-induced iNOS expression. Furthermore, treatment with CoPP IX also abrogated LPS/IFN-γ-induced nitric oxide production (Figure 5E). We further studied the signaling pathways involved in regulatory effects of fisetin on HO-1 in microglia. Treatment with p38 (SB203580) and PI3 kinase/Akt (LY294002) inhibitors, but not ERK (PD98059) and JNK (SP600125) inhibitors effectively antagonized fisetin-induced HO-1 expression (Figure 6A). As shown in Figure 6B,C, incubation with p38 or PI3 kinase/Akt inhibitors reduced fisetin-induced HO-1 expression concentration-dependently. As shown in Figure 6D,E, fisetin increased p38 and Akt activation in a time-dependent manner as well. These results demonstrate that fisetin-induced anti-neuroinflammation may be regulated by HO-1 signaling pathway.

### 2.3. Fisetin Inhibits Microglial Activation in a Mouse Model

**Figure 7:**
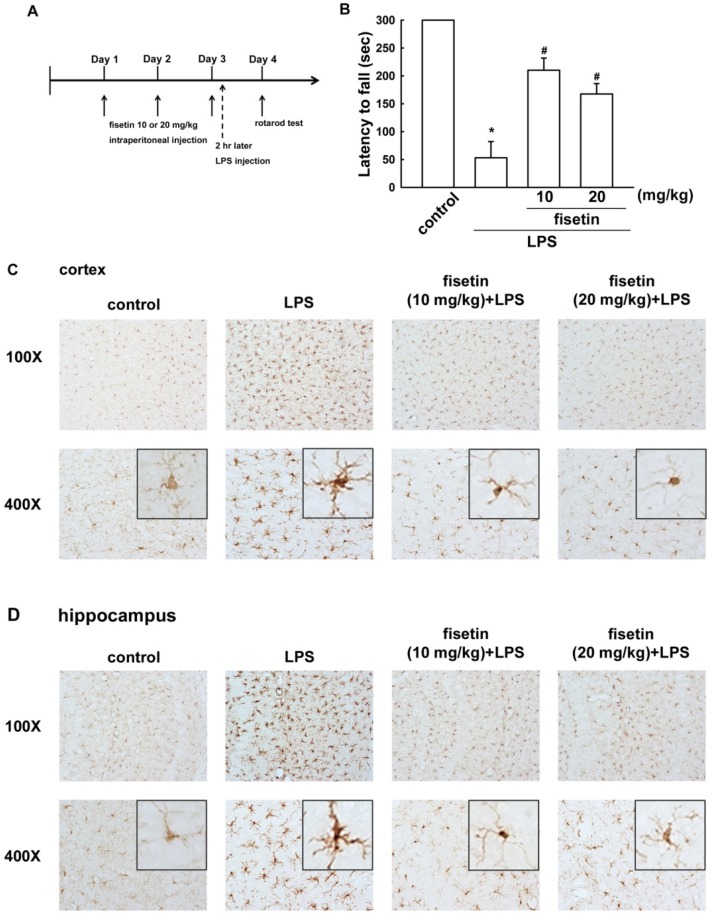
Fisetin prevents LPS-induced microglial activation.Mice received intraperitonealinjections of fisetin at concentrations of either 10 or 20 mg/kg once per day for 3 consecutive days. On the third day, fisetin treatment was followed with a single intraperitoneal injection of LPS (20 mg/kg).Motor balance and coordination function were analyzedusing an accelerating rotarod test (**B**). Microglial morphology was visualized by anti-Iba-1 immunolabeling in cortical (**C**) and hippocampal (**D**) regions.

To determine the improvements induced by fisetin treatment on neuroinflammatory responses *in vivo*, we performed a motor behavior test and an immunohistochemical analysis. Mice were continuously administered fisetin for three consecutive days, and were then injected or not with LPS (Figure 7A). Motor balance and coordination function were analyzed using an accelerating rotarod test [34]. As shown in Figure 7B, LPS-treated mice showed a reduced latency in the rotarod test, thus demonstrating motor impairments compared to the control group. However, administration with fisetin significantly ameliorated the motor-impaired effects of LPS-injected mice (Figure 7B). 

The activation of microglia was analyzed morphologically Iba-1 immunoreactivity by immunohistochemical analysis. After LPS injection for 24 h, activated microglial cells were distributed throughout whole mouse brains. The Iba-1 positive immunoreactivity was much stronger in LPS-treated mice, microglial cells with pronounced hypertrophy and enlarger cell bodies compared with the control groups in cortical and hippocampal regions (Figure 7C,D). Similar to the motor behavioral results, microglial activation in the mice brains were effectively attenuated by administration of fisetin (Figure 7C,D).

## 3. Discussion

There were eight major findings of current study: (1) When microglia were exposed to H_2_O_2_, the intracellular ROS was highly increased, which was mitigated by fisetin. (2) Fisetin effectively inhibited ATP-induced increase microglial migratory activity. (3) LPS/IFN-γ- or peptidoglycan-induced NO production were suppressed by treating fisetin to microglia. (4) Fisetin is nontoxic at 1–5 μM and dose-dependently reduced iNOS and IL-1β productions. (5) LPS plus IFN-γ enhanced STAT signaling activation was diminished by treatment with fisetin. (6) HO-1 levels were up-regulated by fisetin treatment dose-dependently. Treatment with HO-1 activator increased HO-1 expression and abrogated LPS/IFN-γ-induced iNOS/NO expressions. (7) Administration with fisetin significantly ameliorated the motor-impaired effects of LPS-injected mice. (8) Microglial activation in the mouse brain was effectively attenuated by administration of fisetin. Accordingly, fisetin was effective in retarding microglial activation. Fisetin potently inhibits oxidative reactions and proinflammatory responses in microglia, probably via promoting up-regulation of an endogenous antioxidant HO-1 expression.

Cytokines are important mediators involved in immune, inflammatory, and immunomodulatory functions [15]. Although inflammatory responses regulate normal functions of neuronal cells, microglial activation must be tightly control to avoid exaggerated nenurotoxicity [6,13]. Bacterial meningitis is the most frequently fatal infection in CNS, which results in significant neurological sequelae [44,45]. In Gram-negative infections, LPS is a well-known activator of microglia. Peptidoglycan, a major component of the Gram-positive bacterium cell wall, activates microglia and induces productions of chemokines and cytokines [42,43,46]. STAT1 is a key inflammatory signaling molecule to regulate iNOS expression in microglial cells and macrophages [47,48]. Activated microglial cells migrate to the neuronal injury sites and express inflammatory cytokines which aggravate neuronal damage [7,8,9,10]. In the present study, fisetin effectively inhibited microglial cell migration induced by ATP, and inflammatory cytokine expressions induced by both LPS/IFN-γ and peptidoglycan stimulations.

Classical activation of macrophages/microglia by microbial compounds or pro-inflammatory cytokines yields a phenotype, which linked to neuroinflammation and neurotoxicity [49,50]. Otherwise, alternative activation of macrophages/microglia leads to anti-inflammatory phenotype [51,52,53,54], which generates neuronal growth factors as well as anti-inflammatory cytokines, thus contributing to neuroprotection [55,56,57,58]. Therefore, induction of alternative activation of macrophages/microglia and increases in anti-inflammatory cytokines may provide a novel strategy for anti-neuroinflammation and neuroprotection. HO-1 is known to be a potent anti-oxidative enzyme, the key mediators participating in signal transduction mechanisms remain to be fully identified. It has been reported that HO-1 may as a therapeutic target in neurodegenerative diseases and brain infections [59]. Our results support previous reports showing that HO-1 is a critical regulator of NO production in numerous cells [60,61]. Specifically, recent reports have shown that PI-3 kinase/Akt and p38 signaling pathways regulate HO-1 expression in various cells [62,63]. Again, our results from the microglial cells support the role of these pathways in fisetin-induced HO-1 expression. Specifically, fisetin activates Akt and p38 phosphorylation and the blockade of PI-3 kinase/AKT and p38 pathways antagonizes fisetin-induced HO-1 expression.

Intraperitoneal injection of LPS is a useful method for investigating inflammation-caused motor and coordination function deficits in rodent model. Peripheral inflammation exacerbates brain cytokine expression, leading to neurodegeneration [64], motor impairment [65], and cognitive impairment [66]. A recent study has shown that intraperitoneal injection of LPS induced progressive motor impairment from 4 to 24 h [65]. It has also been reported that peripheral LPS injection induced microglial activation and motor impairment [67]. In current study, we also performed an intraperitoneally injection of LPS, which caused a shorter latency on the rotarod test and microglia activation significantly in cortex and hippocampus; however, treatment with fisetin significantly alleviated these motor-impaired effects and microglia activation.

## 4. Experimental

### 4.1. Reagents and Antibodies

Recombinant murine IFN-γ was purchased from PeproTech (Rocky Hill, NJ, USA). LPS from *Escherichia coli* Serotype 055:B5, SB203580, PD98059, SP600125 and LY294002 were obtained from Sigma-Aldrich (St. Louis, MO, USA). Peptidoglycan from *Staphylococcus aureus* was purchased from Fluka (Buchs, Switzerland). The antibody against ionized calcium binding adaptor molecule 1 (Iba 1) was purchased from Wako Pure Chemical Industries (Osaka, Japan). Primary antibodies against Akt, β-actin, phosphorylated Akt, STAT1 and p38 were purchased from Santa Cruz Biotechnology (Santa Cruz, CA, USA). HO-1 antibody was purchased from StressGen Biotechnologies (San Diego, CA, USA). Primary antibodies against phosphorylated p38 and STAT-1 phosphorylated at Tyr^7^^01^ were purchased from Cell Signaling and Neuroscience (Danvers, MA, USA). The primary antibody against iNOS was purchased from BD Transduction Lab (Lexington, KY, USA).

### 4.2. Cell Culture

The murine microglial cell BV-2 was generated from infecting primary microglial cell culture with a v-raf/v-myc oncogene carrying a retrovirus, and the cells retain the morphological, phenotypical, and functional properties of primary microglial cells [68]. Cells were cultured in DMEM (Gibco, Grand Island, NY, USA) with 10% FBS at 37 °C, incubated in a humidified atmosphere consisting of 5% CO_2_ and 95% air.

### 4.3. Animals

The animal experiments were accordance with the Animal Care and Use Guidelines of the China Medical University (Taichung, Taiwan). Eight-week-old male ICR mice were purchased from the National Laboratory Animal Center (Taipei, Taiwan). The mice were housed in a temperature- and humidity-controlled environment, and given free access to foods and water, and acclimated to their environment for at least 7 days before conducting the experiments.

### 4.4. Tissue Preparation and Immunohistochemistry

Mice received an intraperitoneal injection of saline or 20 mg/kg LPS. Twenty-four hours later, mice were deeply anesthetized with chloral hydrate and transcardially perfused with 10% formaldehyde. These brains were sliced coronal serial sections (30 μM) using a freezing sliding microtome cryostat (CM305S, Leica, Microsystems; Wetzlar, Germany). Brain sections were quenched by endogenous peroxidases with hydrogen peroxide, blocked by nonspecific binding with goat serum, permeabilized with Triton X-100, and then incubated with a primary antibody against Iba-1. Following incubation with a biotin-conjugated secondary antibody, the sections were incubated with an avidin-biotin complex (Vector Laboratories, Burlingame, CA, USA), and labeling was visualized with diaminobenzidine. The cerebral cortex and hippocampus were digitally captured at 200× magnification using a light microscope.

### 4.5. Reactive Oxygen Species (ROS) Assay

The procedure of ROS assay was according to our previous studies. Production of ROS was assessed oxidation of specific probes with 2',7'-dichlorodihydrofluorescein diacetate (H_2_DCFDA) by using flow cytometry. Cells were incubated to H_2_DCFDA (10 μM) for 30 min at 37 °C, and then stimulated with hydrogen peroxide. The fluorescence intensity was measured with an excitation filter of 488 and 525 nm emission wavelengths.

### 4.6. Western Blot Analysis

Western blotting was performed according to our previous report [69,70]. Briefly, cells were lysed in a homogenization buffer and equal amounts of the samples were loaded in a SDS-PAGE membrane. The membranes were probed with a primary antibody and then incubated with a peroxidase-conjugated secondary antibody. The blots were developed by a western chemiluminescent HRP substrate (Millipore, Billerica, MA, USA) and visualized by using Fuji medical X-ray film (Fujifilm, Tokyo, Japan). The blots were then stripped by using a stripping buffer [71] and re-probed anti-β-actin antibody to as a loading control.

### 4.7. Migration Assay

*In vitro* migration assay was performed using Costar Transwell inserts (pore size: 8 μm; Corning, Albany, NY, USA) in 24-well plates as described previously [72]. Approximately 1 × 10^4^ cells in 200 μL of medium were placed in the upper chamber, and the same medium containing ATP was placed in the lower chamber [34,73]. Before performing the migration assay, cells were pre-treated for 60 min with fisetin followed by treatment with ATP during the 24-h migration assay (incubated at 37 °C in 5% CO_2_). After the 24-h assay, the cells were stained with 0.05% crystal violet and 2% methanol. Non-migratory cells on the upper surface of the filters were removed by wiping with a cotton swab. Cell number was counted in five random fields per well under a microscope at 200× magnification. Images of migratory cells were observed and acquired using a digital camera and light microscope.

### 4.8. Quantitative Real-Time PCR

Quantitative real-time PCR was performed according to our previous report [35,38]. Briefly, quantitative real-time PCR using SYBR Green Master Mix was performed with StepOne Plus System (Applied Biosystems, Singapore). After incubation at 50 °C for 2 min and 95 °C for 10 min, the PCR was performed as follows: 40 cycles at 95 °C for 10 s and 60 °C for 1 min. The threshold was set above the non-template control background and within the linear phase of target gene amplification to calculate the cycle number at which the transcript was detected (denoted as CT).

### 4.9. Nitric Oxide Assay

Nitric oxide production was analyzed by measuring nitrite content in culture media as described in our previous report [46]. Briefly, the media were determined by a colorimetric assay with a Griess reaction, the absorbance was determined at 550 nm using a microplate reader (Thermo Scientific, Vantaa, Finland).

### 4.10. 3-(4,5-Dimethylthiazol-2-yl)-2,5-diphenyltetrazolium Bromide (MTT) Assay

Cell viability was determined using the MTT assay, the procedure was according to previous reports [74]. After treatment with fisetin for 24 h, cell culture media were removed. MTT (0.5 mg/mL) was added to each culture well and the mixture was incubated for 2 h at 37 °C. The MTT reagent was removed and washed with PBS for several times. DMSO (200 μL per well) was added to dissolve formazan crystals, absorbance was determined at 550 nm using a microplate reader (Thermo Scientific, Vantaa, Finland).

### 4.11. Statistical Analyses

The values are reported as mean ± S.E.M. Statistical analyses for two groups were performed using Student’s *t-*test. The difference was determined to be significant if the *p* value was <0.05.

## 5. Conclusions

The results of current study suggest that fisetin exerts both anti-oxidative and anti-inflammatory activities may be suggested as a promising strategy in the treatment of neurodegenerative diseases.
